# The prenylated pUS2 protein of pseudorabies virus contributes to phosphorylation of connexin 43 and suppression of gap junctional intercellular communication

**DOI:** 10.1128/jvi.00773-26

**Published:** 2026-06-18

**Authors:** Alexander Tishchenko, Benjamin De Boeck, Cliff Van Waesberghe, Fien van Raemdonck, Walter Fuchs, Barbara G. Klupp, Thomas C. Mettenleiter, Oliver Vickman, Gregory A. Smith, Herman W. Favoreel

**Affiliations:** 1 Department of Translational Physiology, Infectiology and Public Health, Faculty of Veterinary Medicine, Ghent University, Merelbeke, Belgium; 2 Institute of Molecular Virology and Cell Biology, Friedrich-Loeffler-Institut, Greifswald-Insel Riems, Germany; 3 Department of Microbiology-Immunology, Feinberg School of Medicine, Northwestern University, Chicago, Illinois, USA; Dartmouth College Geisel School of Medicine, Hanover, New Hampshire, USA

**Keywords:** pseudorabies virus, gap junctions, US2, prenylation, ERK1/2, alphaherpesvirus, immune evasion

## Abstract

Gap junctional intercellular communication (GJIC) enables the direct transfer of signaling molecules between neighboring cells and plays an important role in innate antiviral immunity by facilitating bystander defense responses. We previously showed that the alphaherpesvirus pseudorabies virus (PRV) suppresses GJIC through ERK1/2-mediated phosphorylation and degradation of the main gap junction (GJ) protein connexin 43 (Cx43), triggered by the viral tegument protein pUL46. Here, we identify the prenylated viral protein pUS2 as a second important regulator of this process during PRV infection. Using the attenuated PRV vaccine strain Bartha K61 and a panel of isogenic deletion mutants, we show that pUS2 is required for efficient Cx43 phosphorylation and GJIC suppression but is dispensable for ERK1/2 activation itself. We further demonstrate that this function of pUS2 depends on its C-terminal CAAX prenylation motif. A prenylation-deficient pUS2 mutant fails to localize ERK1/2 to the plasma membrane and, likely as a consequence, does not induce efficient Cx43 phosphorylation or GJIC downregulation. Our data support a model in which PRV employs a two-step strategy to suppress GJIC in infected cells: pUL46 triggers ERK1/2 activation, while newly expressed, prenylated pUS2 redirects the activated kinase to the plasma membrane to facilitate phosphorylation of Cx43. This work reveals how PRV exploits the spatial control of host kinase signaling to suppress gap junction intercellular communication.

**IMPORTANCE:**

Gap junctions (GJs) constitute key communication channels between cells in multicellular organisms. GJs allow the intercellular transfer of different small biomolecules, including messengers involved in the innate and adaptive antiviral response. We showed earlier that, during infection of epithelial cells with the porcine alphaherpesvirus pseudorabies virus (PRV), the viral tegument protein pUL46 triggers activation of ERK1/2, which in turn leads to phosphorylation of the major gap junction protein connexin 43 (Cx43) and closure of GJs. In the current report, we show that a second PRV protein, pUS2, contributes to Cx43 phosphorylation and GJ closure, likely by recruiting ERK1/2 to the plasma membrane. This function of pUS2 depends on its CAAX prenylation motif. The current data suggest that PRV-mediated Cx43 phosphorylation and GJ closure occur via a two-step process, involving the viral pUL46 and pUS2 proteins, and point to GJ modulation as a possible target for antiviral strategies.

## INTRODUCTION

Gap junctions (GJs) enable the direct exchange of ions, metabolites, and signaling molecules between adjacent cells, and are essential for tissue homeostasis and coordination of cellular responses to external stimuli (1, 2). Gap junctions are composed of members of the connexin protein family, with connexin 43 (Cx43) being the most abundant and well-characterized member (1, 2). Besides allowing neighboring cells to operate as a functional syncytium, in recent years, gap junctional intercellular communication (GJIC) has emerged as a pillar in both the intrinsic and adaptive immune response, enabling the transfer of immunogenic peptides (3) and antiviral messengers such as cyclic guanosine monophosphate-adenosine monophosphate (cGAMP) (4) and 2′−5′ oligoadenylate (2-5A) (5) that are synthesized in response to viral DNA or RNA, respectively. Therefore, and not surprisingly, viruses across diverse DNA and RNA families have evolved independent mechanisms that target GJIC and connexin proteins to procure a more permissive environment for replication and spread (6).

The gating of GJ channels is fine-tuned by an intricate interplay between protein-protein interactions (7), connexin turnover (8, 9), and post-translational modifications (PMTs) of connexins (10). Among these mechanisms, phosphorylation of the major GJ protein Cx43 plays a central role in channel gating, trafficking, and degradation (11, 12).

We have recently shown that wild-type (WT) pseudorabies virus (PRV), a porcine alphaherpesvirus, actively suppresses GJIC in infected epithelial cells (13). This suppression is mediated by PRV-induced activation of the host ERK1/2 kinase via the tegument pUL46 protein, which leads to robust phosphorylation and subsequent degradation of the main GJ protein Cx43. Importantly, we found that PRV-induced GJIC inhibition contributes to efficient intercellular virus spread, possibly by suppressing the transfer of antiviral secondary messengers that would trigger a bystander antiviral effect in neighboring cells, such as cGAMP (13). This mechanism suggests a deliberate strategy by PRV to isolate infected cells from their neighbors, thereby potentially limiting immune surveillance or the spread of host-derived antiviral signals. Although we identified pUL46 as a key driver of ERK1/2 activation during PRV infection, how activated ERK1/2 is directed toward the plasma membrane to enable efficient phosphorylation of Cx43 during infection has remained unresolved.

pUS2 is a conserved tegument protein found across most alphaherpesviruses, with the exception of varicella–zoster virus (14). The pUS2 protein is expressed with early kinetics, and the C-terminal CAAX motif of PRV pUS2 undergoes prenylation, which localizes the protein to cellular membranes, including the plasma membrane (15). pUS2 interacts with activated ERK1/2 and can recruit the kinase to the plasma membrane when expressed ectopically (16, 17). However, the functional significance of the pUS2-ERK1/2 interaction during viral infection remains unclear, and whether the membrane targeting of ERK1/2 by pUS2 contributes to any defined viral process is not established.

In the current study, we report that pUS2 contributes to Cx43 phosphorylation and suppression of GJIC in PRV-infected cells, and that this function of pUS2 depends on its prenylation. These data reveal that efficient PRV-induced Cx43 phosphorylation and GJIC suppression not only depend on pUL46-induced ERK1/2 activation but also on the expression of prenylated pUS2. We propose that pUS2-mediated recruitment of pUL46-activated ERK1/2 to the plasma membrane allows ERK1/2 to dock in close proximity to Cx43, facilitating its phosphorylation and the closure of GJs.

## RESULTS

### The US deletion in the genome of the PRV vaccine strain Bartha K61 contributes to its inability to trigger Cx43 phosphorylation or downregulate GJIC

The attenuated PRV strain Bartha K61 (from here on referred to as Bartha) is widely used in PRV vaccination and eradication programs. This strain was generated by serial passaging of a field strain in non-natural host cells (18, 19). As a result, its genome has accumulated several mutations that led to attenuation in the natural host. In particular, the Bartha strain contains a large deletion in the unique short (US) region of the genome, resulting in complete or partial deletion of four viral genes: US2 (encoding the viral tegument protein pUS2), US7 (encoding glycoprotein I, gI), US8 (encoding glycoprotein E, gE), and US9 (encoding the membrane protein pUS9) (20, 21). In addition, the mutations accumulated within the Bartha genome resulted in reduced incorporation of several tegument proteins into the Bartha virions, including pUL46 (22).

**Fig 1:**
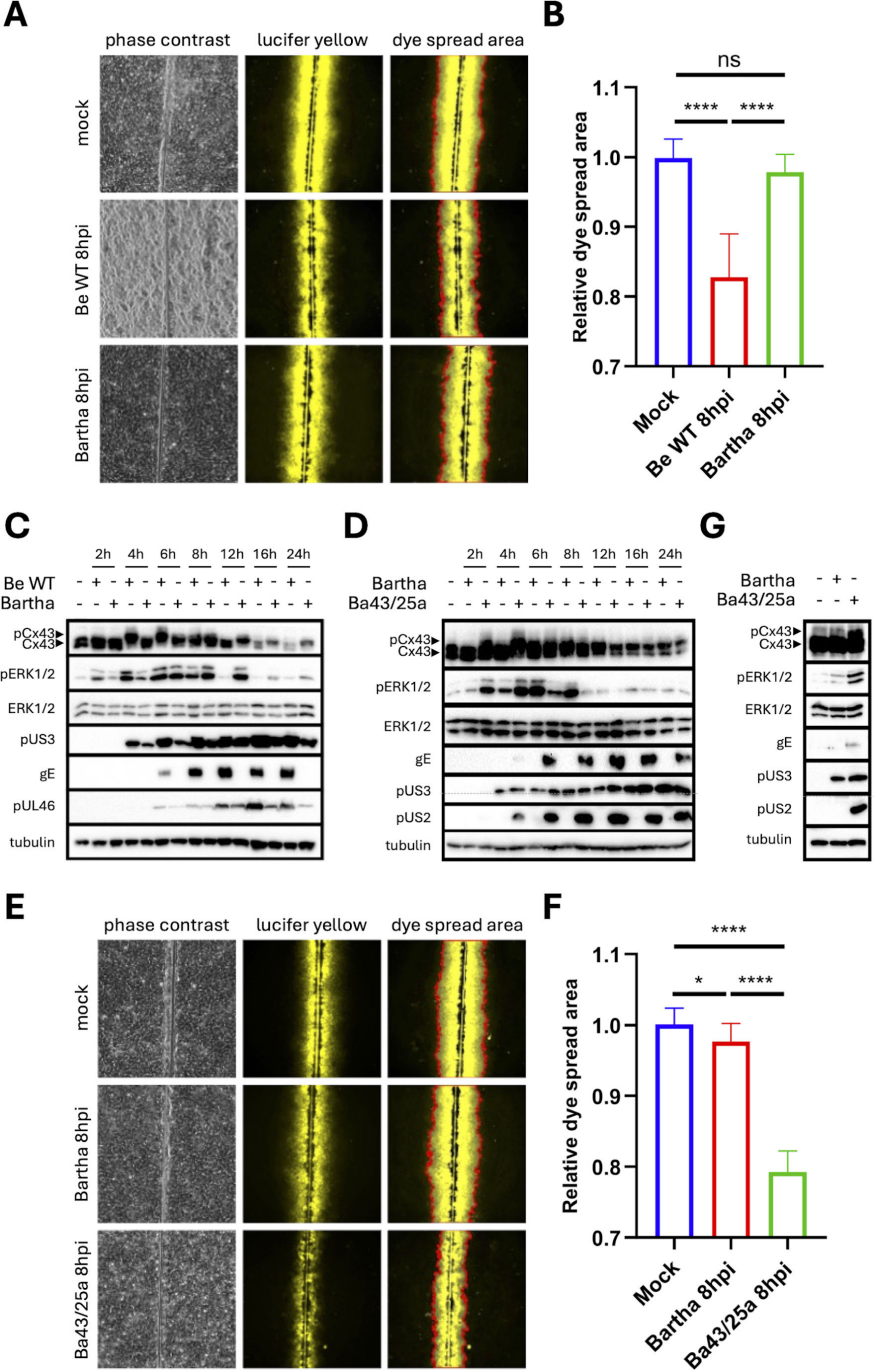
The US genomic region that is absent in PRV Bartha contributes to Cx43 phosphorylation and suppression of GJIC. (**A**) Representative images of SL-DT assays performed in WB-F344 cells that were mock-infected or infected for 8 h with WT PRV strain Becker (Be) or the vaccine strain Bartha (MOI, 10 PFU/cell). (**B**) Quantitative analysis of SL-DT assays as shown in panel A. Dye spread area was normalized to dye spread in mock-infected cells (set to 1). Graphs represent mean and standard deviations of three independent repeats (ns, not significant; **** *P* ≤ 0.0001). (**C**) Western blot analysis of Cx43 phosphorylation and ERK1/2 activation during a time-course assay in ST cells infected with WT PRV strain Becker or the vaccine strain Bartha (MOI, 10 PFU/cell) from 0 to 24 hpi. (**D**) Western blot analysis of Cx43 phosphorylation and ERK1/2 activation during a time-course assay in ST cells infected with the PRV vaccine strain Bartha or an isogenic strain in which the US deletion was restored (Ba43/25a) (MOI, 10 PFU/cell) from 0 to 24 hpi. (**E**) Representative images of SL-DT assays performed in WB-F344 cells that were mock-infected or infected for 8 h with PRV vaccine strain Bartha or the US deletion rescue strain Ba43/25a (MOI, 10 PFU/cell). (**F**) Quantitative analysis of SL-DT assays as shown in panel E. Graphs represent mean and standard deviations of three independent repeats (* *P* ≤ 0.05, **** *P* ≤ 0.0001). (**G**) Western blot analysis of Cx43 phosphorylation and ERK1/2 activation in WB-F344 cells infected with the PRV vaccine strain Bartha or an isogenic strain in which the US deletion was restored (Ba43/25a) (MOI, 10 PFU/cell) at 4 hpi. All Western blots shown in this figure are representative examples from three independent repeats of each experiment.

To assess whether the attenuated Bartha strain suppresses GJIC similarly to WT PRV, scrape loading-dye transfer (SL-DT) assays were performed on mock-, WT PRV strain Becker-, and Bartha-infected WB-F344 cells (the cell type used for SL-DT assays). We observed that, unlike WT PRV, infection with Bartha did not or only very slightly reduce GJIC (Fig. 1A and B). Since we reported earlier that WT PRV-induced suppression of GJIC was caused by ERK1/2-mediated Cx43 phosphorylation, we assessed the phosphorylation status of ERK1/2 and Cx43 in Bartha-infected cells. A time-course infection assay showed that, in comparison with WT PRV-infected cells, infection of cells with the Bartha strain results in delayed and decreased ERK1/2 activation and impaired Cx43 phosphorylation (Fig. 1C).

As mentioned, one notable characteristic of the Bartha genome is a large deletion in the US region resulting in the absence of pUS2, gE, gI, and pUS9 (20, 21). To address whether the lack of this US region is involved in the impaired Cx43 phosphorylation observed in Bartha-infected cells, a time-course assay was performed using the Bartha strain and a Bartha-derivative (Ba43/25a) in which the US deletion is repaired (23). Interestingly, in contrast to infection of cells with the parental Bartha strain, infection of cells with Ba43/25a resulted in robust Cx43 phosphorylation (Fig. 1D), indicating that proteins encoded in the large US deletion in Bartha are responsible for its impaired Cx43 phosphorylation.

We then assessed whether repairing the US deletion in Bartha rescues GJIC inhibition. To this end, SL-DT assays were performed in WB-F344 cells infected for 8 h with Bartha or Ba43/25a. We observed that, unlike Bartha, Ba43/25a suppressed GJIC (13) (Fig. 1E and F). As an additional control, we confirmed that infection with Bartha caused decreased ERK1/2 activation and Cx43 phosphorylation in the WB-F344 cells that are used for SL-DT assays, and that both phenotypes were rescued in cells infected with the Ba43/25a Bartha-derivative (Fig. 1G).

Overall, these data show that the US deletion in Bartha contributes to the impaired ability of Bartha to trigger Cx43 phosphorylation and suppression of GJIC, suggesting that at least one of the four affected viral genes in the US region that is deleted in the Bartha genome contributes to suppression of GJIC.

### The pUS2 protein encoded within the US deletion of Bartha contributes to Cx43 phosphorylation

**Fig 2:**
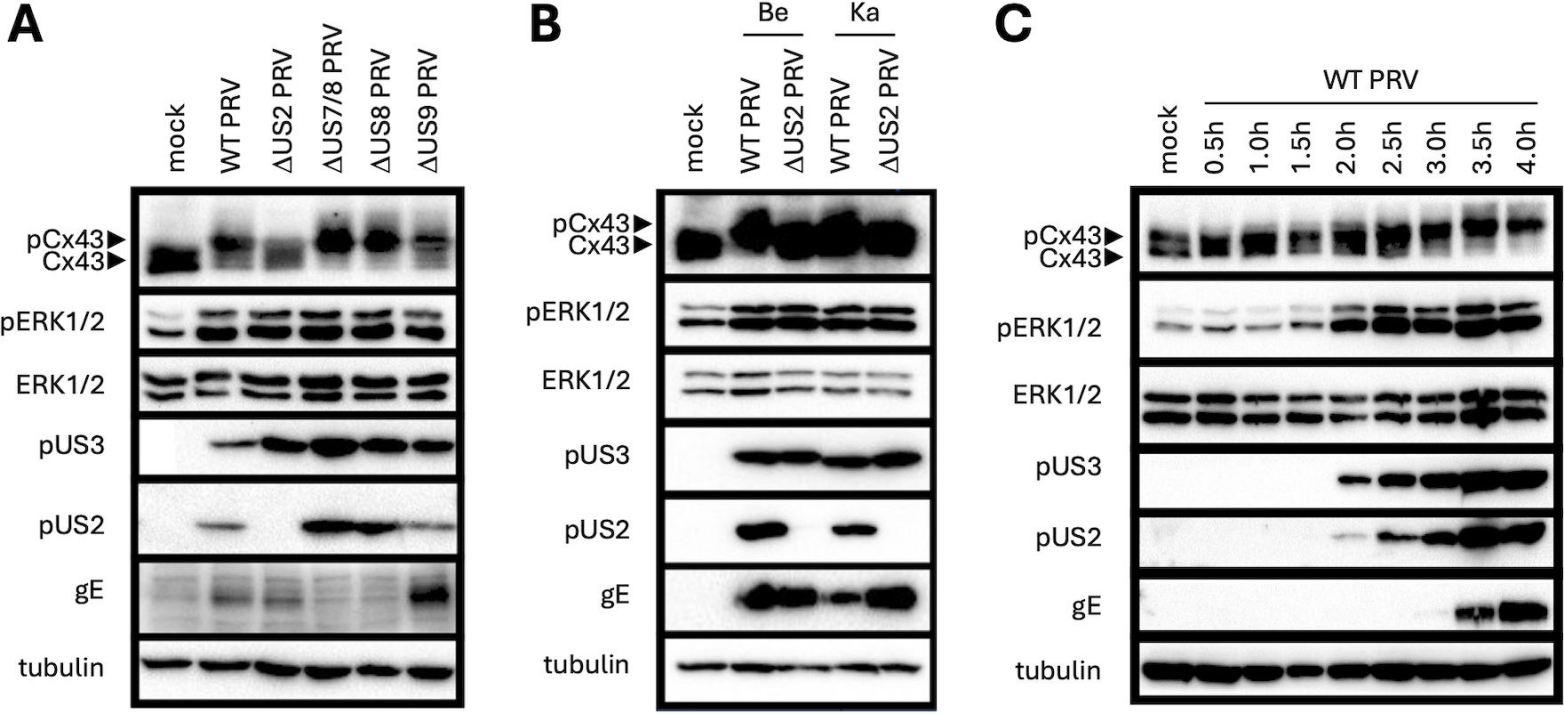
The pUS2 protein promotes Cx43 phosphorylation. (**A**) Western blot analysis of Cx43 phosphorylation and ERK1/2 activation in ST cells infected with WT PRV strain Becker or isogenic mutants in which the US2, US7/8, US8, or US9 genes were deleted (MOI, 10 PFU/cell) at 4 hpi. (**B**) Western blot analysis of Cx43 phosphorylation and ERK1/2 activation in ST cells infected with WT and US2 null PRV mutants in Becker (Be) and Kaplan (Ka) genetic backgrounds at 4 hpi. (**C**) Western blot analysis of Cx43 phosphorylation and ERK1/2 activation during a time-course assay in ST cells infected with WT PRV (MOI, 10 PFU/cell) from 0 to 4 hpi. All Western blots shown in this figure are representative examples from three independent repeats of each experiment.

To assess which of the four viral genes lost by the US deletion in the Bartha genome contributes to Cx43 phosphorylation, infections were performed using WT PRV or isogenic mutants carrying deletions in the US2, US7/US8, US8, or US9 genes. Whereas none of the mutants were defective for ERK1/2 activation, the US2 deletion mutant was uniquely compromised for Cx43 phosphorylation (Fig. 2A). To confirm that the effect of pUS2 is not virus strain-dependent, we assessed ERK1/2 activation and Cx43 phosphorylation in ST cells infected with independently generated US2 deletion mutants (and corresponding wild-type viruses) in the Becker and Kaplan genetic backgrounds (Fig. 2B). Finally, a time-course assay revealed that the start of detectable Cx43 phosphorylation coincided with the start of detectable pUS2 expression, beginning at approximately 2 hpi (Fig. 2C).

### Prenylation of pUS2 is required for PRV-induced Cx43 phosphorylation and for plasma membrane localization of ectopically expressed ERK2

**Fig 3:**
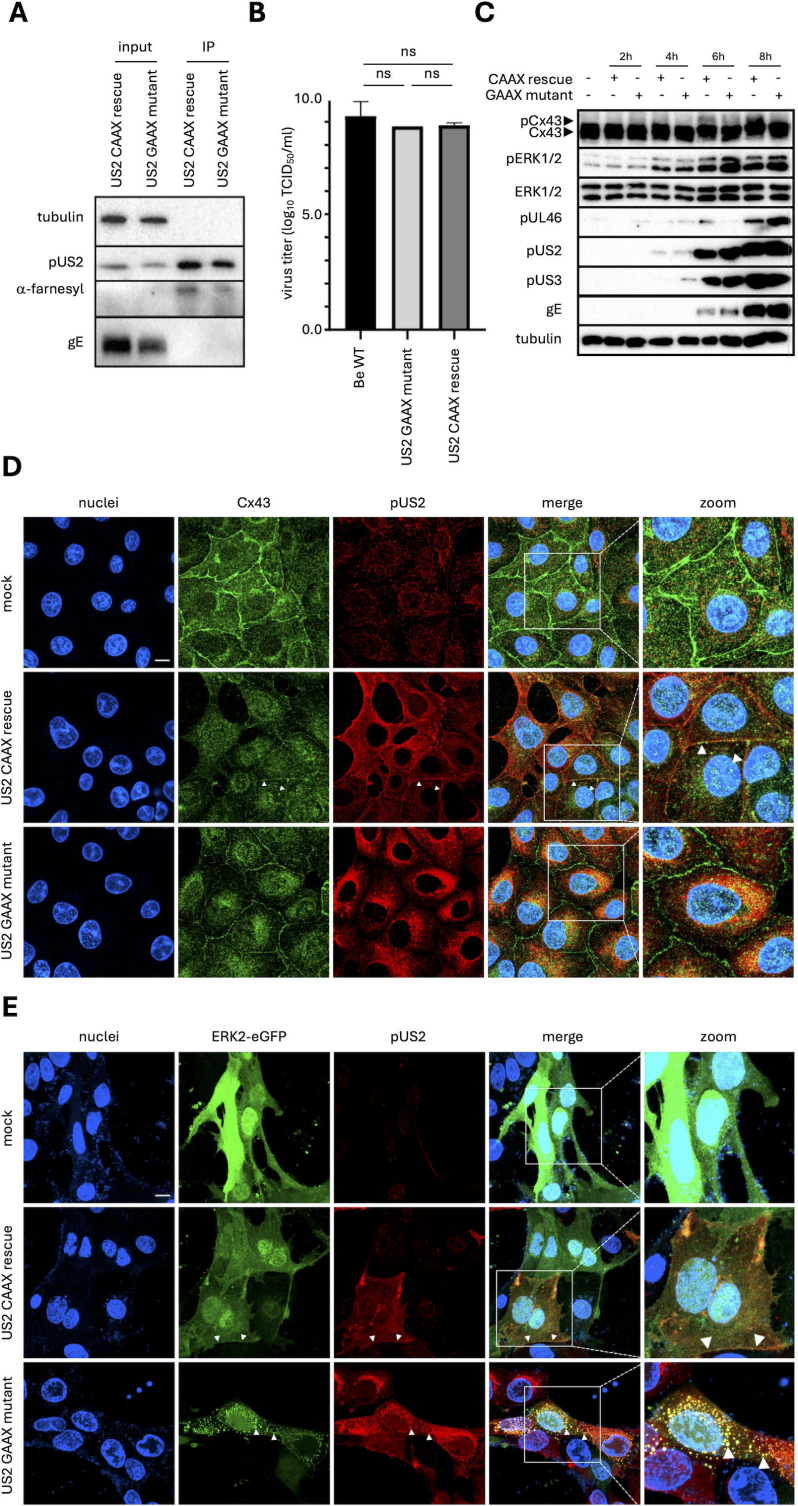
pUS2 prenylation is required for ERK1/2 localization to the plasma membrane and for Cx43 phosphorylation. (**A**) Immunoprecipitation (IP) of pUS2 from ST cells infected with US2 CAAX rescue PRV or US2 GAAX mutant PRV (MOI, 10 PFU/cell, 8 hpi). Input and immunoprecipitated samples were subjected to Western blot analysis using antibodies against tubulin, pUS2, farnesyl, and gE. (**B**) Virus titers of US2 CAAX rescue PRV and US2 GAAX mutant PRV are equivalent. ST cells were infected with either WT PRV strain Becker, US2 CAAX rescue PRV, or US2 GAAX mutant PRV (MOI, 10 PFU/cell). At 24 hpi, supernatants were harvested and titrated. Extracellular virus titers are expressed as TCID_50_/mL on a logarithmic scale. Graphs represent mean and standard deviations of three independent repeats (ns, not significant). (**C**) Western blot analysis of Cx43 phosphorylation and ERK1/2 activation during a time-course assay in ST cells infected with US2 CAAX rescue PRV or US2 GAAX mutant PRV (MOI, 10 PFU/cell) from 0 to 8 hpi. (**D**) Immunofluorescence confocal microscopy of pUS2 and Cx43 localization in ST cells infected with US2 CAAX rescue PRV or US2 GAAX mutant PRV (MOI, 10 PFU/cell) at 8 hpi. Scale bar: 10 μm. (**E**) Immunofluorescence confocal microscopy of ERK2-GFP and pUS2 localization in ST cells transfected with a eukaryotic expression vector encoding ERK2-eGFP, and infected with US2 CAAX rescue PRV or US2 GAAX mutant PRV for 8 h. Scale bar: 10 μm. All Western blots and immunofluorescence assays shown in this figure were taken from one representative assay out of three independent repeats of each experiment.

The pUS2 protein of PRV contains a CAAX prenylation motif at its C-terminus that localizes the protein to the plasma membrane in transfected cells (15). More specifically, PRV pUS2 is thought to be farnesylated (a subtype of prenylation) (15). In addition, pUS2 binds and sequestrates ERK1/2 at the plasma membrane in US2-transfected cells (16, 17). Considering the canonical plasma membrane localization of Cx43 and our observation that pUS2 contributes to Cx43 phosphorylation, we assessed whether pUS2 prenylation was important for its ability to enhance Cx43 phosphorylation. To assess this, a PRV mutant encoding a prenylation-deficient pUS2 protein (C→G mutation of the prenyl-acceptor cysteine in the CAAX motif; hereafter referred to as US2 GAAX mutant PRV) and a corresponding rescue PRV strain in which the mutation in US2 was restored (hereafter referred to as US2 CAAX rescue PRV) were constructed. As a control, pUS2 was immunoprecipitated from ST cells infected with either US2 CAAX rescue PRV or US2 GAAX mutant PRV and subjected to Western blot analysis, which showed a clear anti-farnesylation signal for US2 CAAX rescue PRV, while this was less evident for US2 GAAX mutant PRV (Fig. 3A). Titration assays at 24 hpi (MOI 10) showed that viral replication efficiency of these virus strains was on par with WT PRV (Fig. 3B). A time-course assay in ST cells showed that the US2 CAAX rescue PRV strain triggered potent Cx43 phosphorylation, while the US2 GAAX mutant PRV displayed decreased Cx43 phosphorylation (Fig. 3C). Immunofluorescence assays showed co-localization of pUS2 with Cx43 at the plasma membrane in cells infected with the US2 CAAX rescue PRV, whereas in cells infected with the US2 GAAX mutant PRV, pUS2 appeared to inefficiently localize at the plasma membrane and did not co-localize with Cx43 (Fig. 3D). The inability of GAAX pUS2 to localize at the plasma membrane is in line with what has been previously described in US2 transfection assays expressing wild-type or prenylation-defective mutant pUS2 (15). In addition, in ST cells that were transfected with a plasmid encoding ERK2-GFP and then infected with US2 CAAX rescue PRV, some ERK2-GFP co-localized with pUS2 at the plasma membrane (Fig. 3E). However, in US2 GAAX mutant PRV-infected cells, ERK2-GFP accumulated with pUS2 in the cytosol (Fig. 3E), suggesting that the prenylation and plasma membrane localization of pUS2 promote ERK1/2 localization at the plasma membrane in PRV-infected cells.

These results indicate that prenylation of pUS2 contributes to efficient Cx43 phosphorylation in PRV-infected cells, likely because of its ability to guide activated ERK1/2 to the plasma membrane.

### pUS2 prenylation is required for efficient downregulation of GJIC

**Fig 4:**
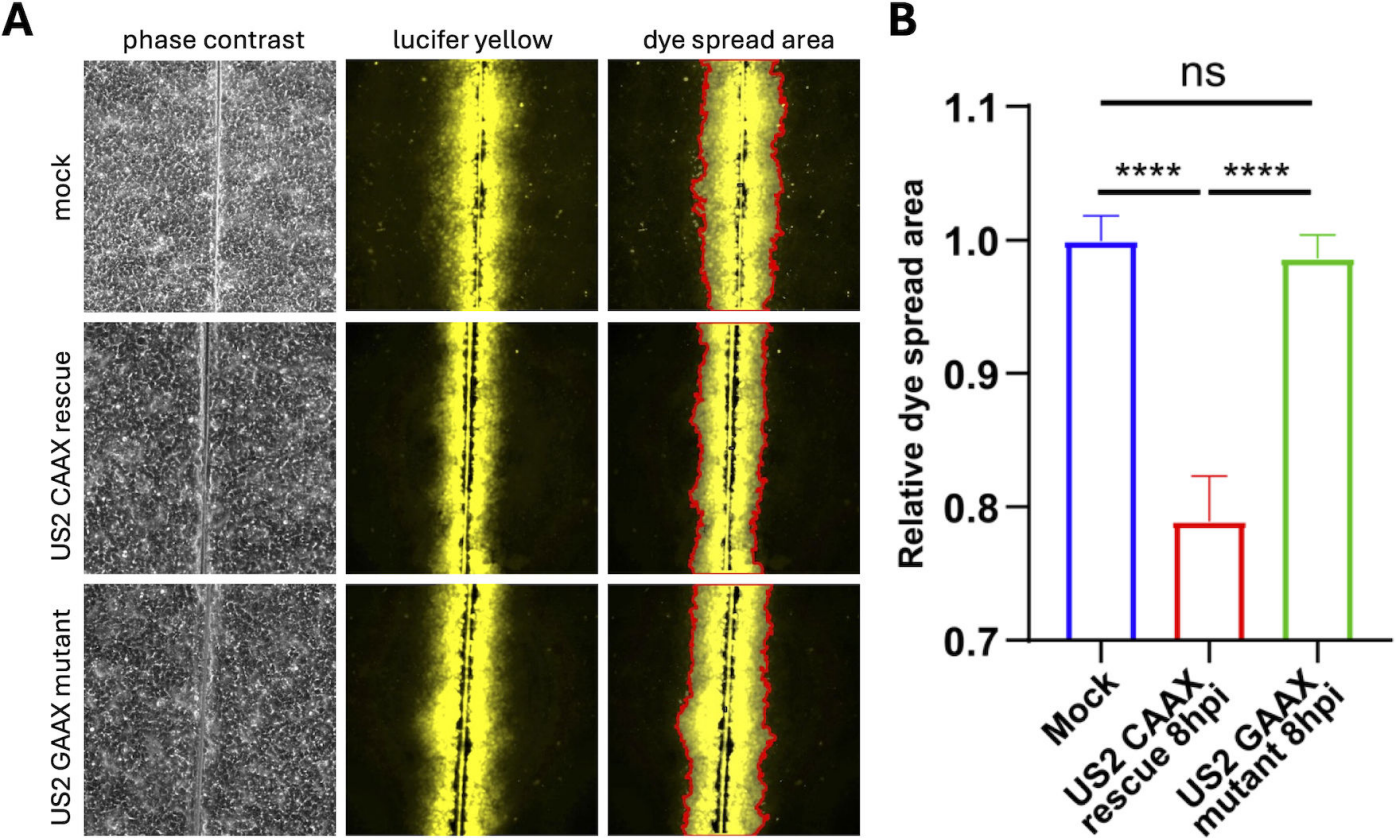
pUS2 prenylation is required for PRV-induced downregulation of GJIC. (**A**) Representative images of SL-DT assays performed in WB-F344 cells that were mock-infected or infected for 8 h with US2 CAAX rescue PRV or US2 GAAX mutant PRV (MOI, 10 PFU/cell). (**B**) Quantitative analysis of SL-DT assays as shown in panel A. Dye spread area was normalized to dye spread in mock-infected cells (set to 1). Graphs represent mean and standard deviations of three independent repeats (ns, not significant; **** *P* ≤ 0.0001).

Considering the importance of pUS2 prenylation in ERK1/2-mediated phosphorylation of Cx43, we investigated whether prenylation of pUS2 also promotes PRV-mediated downregulation of GJIC. To assess this, SL-DT assays were performed on WB-F344 cells infected with either US2 GAAX mutant PRV or US2 CAAX rescue PRV. US2 CAAX rescue PRV-infected cells displayed a significant reduction in GJIC, which was not observed in US2 GAAX mutant PRV-infected cells (Fig. 4A and B). Hence, prenylation of pUS2 antagonizes GJIC.

## DISCUSSION

**Fig 5:**
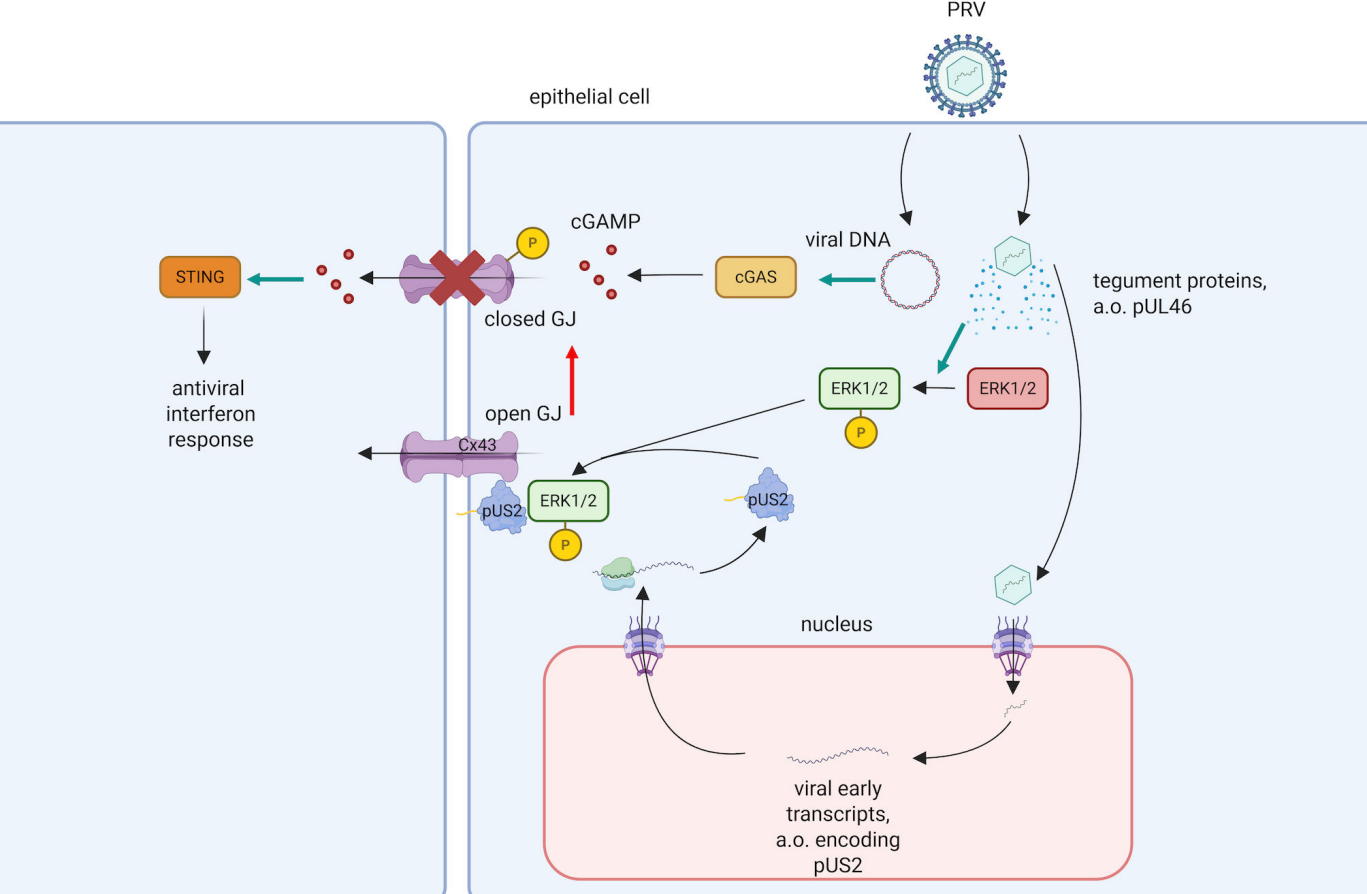
Hypothetical model of PRV-induced ERK1/2-mediated Cx43 phosphorylation and GJIC inhibition. Shortly after PRV infection, pUL46 activates the host ERK1/2 kinase. Upon expression of pUS2, which carries a CAAX prenylation motif, pUS2 sequesters pUL46-activated ERK1/2 to the plasma membrane. Activated ERK1/2 phosphorylates Cx43, leading to closure of GJ channels. Suppression of GJIC boosts viral spread to neighboring cells, likely by interfering with intercellular transfer of antiviral secondary messengers. Figure was generated with BioRender.

GJIC plays an increasingly appreciated role in antiviral immunity by enabling the spread of immune-stimulatory signals between infected and uninfected cells (4, 5). We previously demonstrated that PRV actively suppresses GJIC through ERK1/2-mediated phosphorylation and degradation of Cx43 induced by the tegument pUL46 protein, thereby promoting efficient virus spread (13). In the present study, we identify a second viral protein, pUS2, as a critical factor that facilitates this process, likely by localizing ERK1/2 to the plasma membrane. Our data support a model in which PRV pUL46-induced ERK1/2 activation is coupled with pUS2 recruitment of ERK1/2 to gap junctions at the plasma membrane, thereby promoting Cx43 phosphorylation and GJIC suppression (Fig. 5).

ERK1/2 is a central regulator of cell proliferation, differentiation, and survival (24). More than 600 direct ERK1/2 substrates have been identified, with many additional proteins being phosphorylated indirectly downstream of ERK1/2 signaling (25). Although many of them are nuclear transcription factors, ERK1/2 also targets substrates in the cytosol, including proteins associated with the cytoskeleton and cellular membranes (26). Crucially, ERK1/2 activation does not result in indiscriminate substrate phosphorylation; instead, only a defined subset of substrates is phosphorylated in response to a given stimulus. This selectivity is thought to arise, at least in part, from ERK1/2 scaffolding proteins that impose spatial control over its activity and channel the kinase toward stimulus-appropriate targets, tethering ERK1/2 to specific organelles and biasing its substrate choice (26, 27). Such spatial regulation provides an attractive mechanism by which viruses could redirect ERK1/2 activity toward specific host targets without altering upstream activation.

Although the alphaherpesvirus pUS2 tegument protein is dispensable for viral replication in cell culture, US2 is deleted in several attenuated vaccine strains such as PRV Bartha (28, 29), bovine alphaherpesvirus 1 (BoHV-1) S-IBR-052 (30), and equine alphaherpesvirus 1 (EHV-1) RacH (31); and restoration of US2 substantially contributes to enhanced virulence in *in vivo* and *ex vivo* models (15, 31), pointing to an important role during natural infection. However, early studies failed to assign a clear molecular function to pUS2 homologs. Of note, of the sequenced alphaherpesvirus US2 orthologs, only pUS2 of PRV and BoHV1.2 contain a C-terminal CAAX prenylation motif (15). Nonetheless, despite the lack of a prenylation motif, pUS2 orthologs of HSV-2 and EHV-1 are also membrane-associated via an unclear mechanism (31, 32), suggesting that membrane localization of pUS2 may be biologically relevant for different alphaherpesviruses.

ERK1/2 activation is a common consequence of viral infection and has been documented for a wide range of DNA (33) and RNA viruses (34), including several herpesviruses such as HSV-1 (35, 36), HSV-2 (37), VZV (38), EHV-1 (39), BoHV-1 (40), KSHV (41), HCMV (42, 43), and EBV (44); and is commonly associated with enhanced viral spread and replication (42, 43, 45–51). In the case of PRV, ERK1/2 activation occurs rapidly (13, 17, 52), prior to late gene expression.

Our data place pUS2 downstream of ERK1/2 activation but upstream of Cx43 phosphorylation. ERK1/2 activation occurs normally in US2 null-infected cells, yet phosphorylation of Cx43 is reduced (Fig. 2A and B), indicating that pUS2 does not function as an ERK1/2 activator, in line with previous reports (17). Instead, pUS2 acts as a spatial regulator that facilitates access of activated ERK1/2 to membrane-associated substrates. This conclusion is strongly supported by our assessment of the prenylation-deficient US2 GAAX mutant. Disruption of the C-terminal CAAX motif prevents efficient plasma membrane localization of pUS2 (Fig. 3D), abrogates ERK1/2 recruitment to the plasma membrane (Fig. 3E), and results in reduced Cx43 phosphorylation and failure to downregulate GJIC (Fig. 3C and 4). Importantly, these effects are not due to defects in viral replication, as the US2 GAAX mutant PRV replicates similarly to WT PRV and US2 CAAX rescue PRV strains (Fig. 3B). Thus, pUS2 prenylation is specifically required for the spatial organization of ERK1/2 signaling rather than for viral fitness *per se*. Of interest, the pUS2 incorporated into virions is not prenylated, whereas pUS2 expressed *de novo* during infection is prenylated (15), in line with our time-course assays that expression of pUS2 in infected cells coincides with the onset of Cx43 phosphorylation.

In line with previous assays in US2-transfected cells (15), we found that disruption of the C-terminal CAAX prenylation motif in pUS2 in the PRV genome reroutes the protein from the plasma membrane of infected cells to intracellular vesicular structures. Although the identity of these vesicular structures is currently unclear, it is reasonable to hypothesize that these may be (sorting) endosomal compartments, which represent a common destination for mislocalized membrane proteins (53). In line with this, Clase et al. reported that, at least in US2-transfected cells, these vesicles partly co-localize with microtubules (15), which are known to transport endosomes (54).

As expected, immunoprecipitated pUS2 from US2 CAAX rescue PRV-infected cells showed a strong Western blot signal using an anti-pan-farnesyl antibody while this signal was weak when using US2 GAAX mutant PRV (Fig. 3A). Although the latter signal was weak, it was not absent. Different hypothetical explanations can be given for this observation. Anti-pan-farnesyl antibodies recognize a broadly shared post-translational modification motif rather than a unique linear amino-acid sequence. Consequently, they are intrinsically more prone to cross-reactivity and nonspecific/aspecific signal than antibodies directed against unique linear peptide epitopes (55, 56). Alternatively, the weak anti-farnesyl signal observed upon immunoprecipitation of GAAX mutant pUS2 may indicate that this mutant displays some remaining prenylation. Although mutation of the cysteine residue within the CAAX motif is expected to abolish canonical farnesylation, low-level atypical prenylation at alternative cysteine residues cannot be formally excluded, given the documented flexibility of prenyltransferase substrate recognition (57, 58). Seenthe dramatic shift in intracellular distribution of pUS2 in US2 CAAX rescue PRV-infected cells versus US2 GAAX mutant PRV-infected cells, we believe the latter possibility is unlikely, but cannot be ruled out.

We found that the PRV vaccine strain Bartha fails to suppress GJIC in infected cells and shows impaired ERK1/2 activation and Cx43 phosphorylation (Fig. 1A through C). Restoration of the large US deletion in Bartha fully rescues these phenotypes (Fig. 1D through G), directly linking the US region to ERK1/2-dependent modulation of gap junctions. Future research will reveal to what extent the inability to inhibit GJIC contributes to the attenuated phenotype and/or immunogenicity of the PRV Bartha vaccine. This could aid in the rational design of attenuated virus vaccine strains against other alphaherpesviruses that are unable to trigger GJ closure, thereby contributing to reduced viral spread and possibly to increased innate (4, 5) and adaptive (3) immune responses. Similar approaches where vaccine candidates have been designed with the aim of minimizing the immune evasion capabilities of the pathogen have been widely employed in the past (59–63).

Together, these findings define pUS2 as a virus-encoded spatial regulator of ERK1/2 signaling that uncouples ERK1/2 activation from nuclear signaling and redirects kinase activity to the plasma membrane. By doing so, pUS2 likely biases ERK1/2 substrate selection toward membrane-associated targets such as Cx43. This spatial bias may enhance the efficiency of gap junction closure, revealing a sophisticated strategy to exploit host MAP kinase signaling and suppress GJIC.

## MATERIALS AND METHODS

### Cells and viruses

Swine testicle epithelial (ST) cells (ATCC CRL-1746; *Sus scrofa*, pig) were cultured in modified Eagle’s medium (MEM) supplemented with 10% inactivated fetal bovine serum (FBS), 100 U/mL penicillin, 0.1 mg/mL streptomycin, 50 μg/mL gentamicin, and 1 mM sodium pyruvate (all from Gibco, Thermo Fisher Scientific, Waltham, MA, USA).

Rat liver epithelial cells (WB-F344) (RRID:CVCL_9806, *Rattus norvegicus*, rat) were purchased from Cell Lines Service (CLS) GmbH (Eppelheim, Germany), and cultured in Dulbecco’s modified Eagle’s medium (DMEM) supplemented with 10% FBS, 100 U/mL penicillin, 0.1 mg/mL streptomycin, 50 μg/mL gentamicin, and 1 mM sodium pyruvate (all from Gibco, Thermo Fisher Scientific).

The PRV strains used were Becker WT (64), Becker ΔUS2 (PRV174) (15), Becker ΔUS7/8 (PRV99) (65), Becker ΔUS8 (PRV 91) (66), Becker ΔUS9 (PRV161) (67) (all kindly provided by L. Enquist, Princeton University, USA), NIA-3 WT (68), Kaplan WT (69), Bartha K61 (Bartha) vaccine strain (18), and Bartha rescue strain Ba43/25a (US deletion rescue) (23).

Becker US2 GAAX mutant and rescue viruses were constructed for the current study using the pBecker3 infectious clone (70) maintained in GS1783 *Escherichia coli* (71), employing two-step Red recombination as described previously (72). Briefly, a kanamycin resistance cassette flanked by I-SceI restriction sites was amplified using primers containing homology arms targeting the US2 locus. The cassette was inserted into the pBecker3 BAC by Red recombination, generating a merodiploid intermediate in which the surrounding US2 sequence was partially duplicated and carried the desired coding change. Resolution of this intermediate was achieved by I-SceI–mediated cleavage followed by a second round of Red recombination across the duplicated flanking sequences. In the Becker US2 GAAX mutant (PRV-GS7661), a single C-terminal codon substitution (CTIS→GTIS) was introduced to disrupt the native CAAX motif. This mutation was subsequently reverted (GTIS→CTIS) to generate the Becker US2 CAAX rescue virus (PRV-GS8097).

The Kaplan ΔUS2 deletion mutant was generated independently using Red recombinase–mediated mutagenesis of a BAC clone of the PRV Kaplan genome. The US2 open reading frame of PRV strain Kaplan (PRV-Ka) (69) was first amplified by PCR using Platinum Pfx DNA polymerase (Invitrogen) and cloned into pcDNA3 (Invitrogen). Codons 30–235 of US2 (of a total of 257 codons) were replaced by a kanamycin resistance cassette flanked by flippase recognition target (FRT) sites derived from plasmid pKD13 (73). The modified US2 fragment was then amplified and used for Red recombination in *E. coli* carrying a glycoprotein B (gB)–deleted PRV-Kaplan BAC (pPRV-ΔgB), as described previously (74). The kanamycin resistance cassette was subsequently excised by flippase-mediated recombination in *E. coli*. Infectious virus was reconstituted by cotransfection of the modified BAC with a gB rescue plasmid (pUC-B1BclI) into RK-13 cells, restoring the essential gB gene. The resulting PrV-Kaplan ΔUS2 virus exhibited WT-like replication kinetics in cell culture, and the deletion was confirmed by Southern blotting, PCR, and sequencing of viral genomic DNA.

Virus stocks were grown and titrated by serial dilution on ST cells. Every infection was performed on confluent cell monolayers and always using a multiplicity of infection of 10 plaque-forming units per cell (MOI of 10 PFU/cell).

### Titration assay

ST cell monolayers were infected at a multiplicity of infection of 10 PFU/cell with Becker WT, Becker US2 CAAX rescue, or Becker US2 GAAX mutant PRV. At 2 hpi, the cells were treated with sodium citrate buffer (pH 3.0; 40 mM sodium citrate, 10 mM KCl, and 135 mM NaCl) for 2 min at room temperature (RT) to inactivate remaining infectious virus from the inoculum. At 24 hpi, the supernatants containing infectious progenies were harvested. These were titrated by 1/10 serial dilution assays on ST cells seeded on 96-well plates, performed in quadruplicate. PRV-induced cytopathic effect served as a readout.

### Antibodies for the detection of PRV proteins

The antibodies used for the detection of PRV proteins were mouse anti-gE (1:100 for WB) (75), mouse anti-pUS3 (1:100 for WB) (76) (kindly provided by L. Enquist), goat anti-pUS2 (1:10,000 for WB, 1:500 for IF) (15) (kindly provided by B. Banfield), and rabbit anti-pUL46 (1:100,000 for WB) (77).

### Antibodies for the detection of cellular proteins/modifications

Horseradish peroxidase (HRP)-conjugated mouse anti-α-tubulin (1:2,000 for WB, Abcam, Cambridge, United Kingdom, catalog number ab40742), rabbit anti-Cx43 (1:2,000 for WB, Abcam, catalog number ab11370), rabbit anti-total ERK1/2 (p44/42 MAPK) (1:1,000 for WB, Cell Signaling Technology, Danvers, MA, USA, catalog number #9102), rabbit anti-phospho ERK1/2 (p44/42 MAPK) (Thr202/Tyr204) (1:1,000 for WB, Cell Signaling Technology, catalog number #9101), and rabbit anti-pan-farnesylation antibody (1:1,000 for WB, Abcam, catalog number ab199481).

### Immunoprecipitation

At 16 hpi, cells were lysed in TBS containing 1% Triton X-100 supplemented with cOmplete Mini EDTA-free Protease Inhibitor Cocktail (Roche, catalog number 11836170001). Cell lysates were diluted 1:1 with ultrapure water prior to the addition of the immunoprecipitation antibody (goat anti-pUS2). The lysate-antibody mixture was incubated overnight at 4°C on a rotating wheel. The following day, magnetic Protein A/G beads (Thermofisher, catalog number 88802) were added, and the samples were incubated for an additional 4 h at 4°C with rotation. The beads were then washed five times with wash buffer containing 150 mM NaCl and 50 mM Tris-HCl (pH 7.5). Bound proteins were eluted by the addition of Laemmli sample buffer to the samples and heating at 95°C for 5 min. Immunoprecipitates were analyzed by Western blotting as described previously. To prevent interference from denatured immunoglobulin heavy and light chains derived from the immunoprecipitation antibody, Veriblot detection reagent (Abcam, catalog number ab131366) was used for the detection of pUS2.

### Western blotting assays

Samples were harvested in ice-cold 1× RIPA buffer, made from 10× RIPA buffer (catalog number ab156034, Abcam) diluted in ultrapure water containing protease inhibitor cocktail (cOmplete mini EDTA-free, catalog number 11836170001, Roche, Basel, Switzerland) and phosphatase inhibitor cocktail (PhosStop, catalog number: 4906845001, Roche). Cell lysates were kept at 4°C for 20 min before storage at −20°C. SDS-PAGE and Western blotting procedures were extensively described in reference 78. To detect different proteins with similar molecular weight from the same sample within a single experiment, separate SDS-PAGE assays were performed, and the same volume of sample was loaded on each separate SDS-PAGE gel. The blocking solution was composed of 5% (wt/vol) non-fat dry milk diluted in 0.1% PBS-Tween 20 (PBS-T). However, for the detection of phosphorylated proteins, the blocking buffer used consisted of 5% (wt/vol) bovine serum albumin (BSA) (fraction V, catalog number 1120180100, Sigma-Aldrich, St. Louis, MI, USA) diluted in PBS-T. Blotted PVDF membranes were blocked for 1 h at RT. Primary antibodies diluted in the corresponding blocking buffer were incubated with gentle shaking overnight at 4°C. Prior to the incubation with the secondary antibodies, three washing steps with PBS-T of 10 min each at RT were carried out. HRP-linked secondary antibodies, goat anti-IgG mouse-HRP (1:2,000, catalog number P0447, Dako), goat anti-IgG rabbit-HRP (1:3,000, catalog number P0448, Dako), or rabbit anti-IgG goat-HRP (1:2,000, catalog number P0449, Dako) were diluted in the corresponding blocking solution and incubated at RT for 1 h. After three washing steps, protein bands were visualized by chemiluminescence using a ChemiDoc imaging device (Bio-Rad, Hercules, CA, USA). Depending on the levels of the targeted protein and the antibody sensitivity, ECL Plus substrate (GE Health Care, Chicago, IL, USA) or SuperSignal West Femto maximum sensitivity substrate (Thermo Fisher Scientific) was used.

### Transfections and plasmids

The ERK2-GFP plasmid encoding for rat (*Rattus norvegicus*) ERK2 C-terminally linked to enhanced green fluorescent protein (eGFP) (79) was a kind gift from professor N. W. Bunnett (New York University, NY, USA). As transfection control, the cells were transfected with pCDNA3-eGFP plasmid encoding eGFP (purchased from Addgene, catalog number 13031).

The transfection of subconfluent ST cells (approximately 60% confluency) was performed on 24-well plates using JetPrime (Polyplus, catalog number 101000015) according to the manufacturer’s instructions. At 48 h post-transfection, transfected cells were infected and analyzed.

### Immunofluorescence assays

For pUS2 visualization in ERK2-eGFP-transfected cells, cell monolayers were rinsed with sterile PBS containing calcium and magnesium (+Ca, Mg) prior to fixation using 4% paraformaldehyde for 10 min at RT. Then, monolayers were rinsed twice with PBS (+Ca, Mg). The cells were then permeabilized using 0.1% Triton-X100 in PBS (+Ca, Mg) for 2 min at RT. Then, the samples were rinsed three times (for 3 min each) with PBS (+Ca, Mg) 1% BSA (wt/vol) (this solution was filtered through a 0.45 μm membrane to remove insoluble particles). Primary goat anti-pUS2 antibody was diluted 1:500 in PBS (+Ca, Mg) 1% BSA (wt/vol), and centrifuged for 1 min at 13,000 rpm to remove particulates. The supernatant from this centrifugation step was used to incubate the samples for 1 h at RT in a humidified chamber. After primary antibody incubation, the samples were rinsed three times (for 3 min each) with PBS (+Ca, Mg) 1% BSA (wt/vol). The secondary antibody was donkey anti-goat IgG (Alexa Fluor 594) (Thermo Fisher Scientific, catalog number A-11058) and was diluted in PBS (+Ca, Mg) 1% BSA (wt/vol). The secondary antibody solution was centrifuged at 10,000 rpm for 3 min, and the supernatant was used to incubate the cells for 1 h at RT in a humidified chamber protected from light. After the secondary antibody incubation, the samples were rinsed three times (for 3 min each) with PBS (+Ca, Mg) 1% BSA (wt/vol). Then, the nuclei were counterstained with Hoechst 33342 (Thermo Fisher Scientific, catalog number H3570) diluted 1:1,000 in PBS (+Ca, Mg) for 10 min at RT. Finally, the cells were rinsed two more times with PBS (+Ca, Mg) and then mounted on microscope slides using glycerine-DABCO.

For the co-visualization of Cx43 and pUS2, cell monolayers were rinsed with sterile PBS containing calcium and magnesium (+Ca, Mg) prior to fixation using ice-cold (−20°C) methanol for 20 min at −20°C. Then, the samples were rinsed three times with PBS (+Ca, Mg) and blocked with PBS (+Ca, Mg) containing 3% BSA (wt/vol) (filtered through a 0.45 μm membrane) for 1 h at RT. Monolayers were then rinsed twice with PBS (+Ca, Mg). For the immunostaining, the primary antibodies rabbit anti-Cx43 and goat-anti-pUS2 were diluted 1:3,000 and 1:500 in PBS (+Ca, Mg) containing 1% BSA (wt/vol) and centrifuged for 1 min at 13,000 rpm to remove particulates. The supernatant from this centrifugation step was used to incubate the samples overnight at 4°C. Following this step, primary antibody solution was removed, and the cells rinsed three times (for 5 min each) with PBS (+Ca, Mg). The secondary antibodies used were donkey anti-rabbit IgG conjugated to Alexa Fluor 488 for pUS2 detection (Thermo Fisher Scientific, catalog number A-21206) and donkey anti-goat IgG conjugated to Alexa Fluor 594 for pUS2 detection (Thermo Fisher Scientific, catalog number A-11058). Secondary antibodies were diluted 1:200 in PBS (+Ca, Mg) and centrifuged for 3 min at 10,000 rpm to remove particulates. The supernatant from this centrifugation step was used to incubate the samples for 1 h at 37°C. After the secondary antibody incubation, the samples were rinsed three times (for 5 min each) with PBS (+Ca, Mg). The nuclei were then counterstained with Hoechst 33342 (Thermo Fisher Scientific, catalog number H3570) diluted 1:1,000 in PBS (+Ca, Mg) for 10 min at RT. Finally, the cells were rinsed two more times with PBS (+Ca, Mg) and then mounted on microscope slides using glycerine-DABCO.

All pictures shown were taken using a Zeiss LSM900 Axio Imager M2 confocal microscope (Zeiss, Oberkochen, Germany). The resulting images were analyzed using ImageJ imaging software (NIH, USA).

### Scrape loading-dye transfer (SL-DT) assay

The SL-DT assay was performed as described by Sovadinová et al. (80). The complete experimental procedure for the SL-DT assay in WB-F344 is described in reference 13.

### Statistical analysis

Statistical analyses were performed using Prism 6 (GraphPad Software). Statistical differences among the experimental groups were determined using one-way ANOVA assays with Tukey’s multiple comparisons test.

## Data Availability

All data supporting the findings of this study are available from the corresponding author upon reasonable request.
